# Copper/Zinc Superoxide Dismutase in Human Skin: Current Knowledge

**DOI:** 10.3389/fmed.2020.00183

**Published:** 2020-05-12

**Authors:** Giovanna G. Altobelli, Susan Van Noorden, Anna Balato, Vincenzo Cimini

**Affiliations:** ^1^Department of Advanced Biomedical Sciences, Medical School, “Federico II” University of Naples, Naples, Italy; ^2^Department of Histopathology, Imperial College London, Hammersmith Hospital, London, United Kingdom

**Keywords:** Cu/Zn superoxide dismutase, human skin, immunochemistry, skin tumors, ROS

## Abstract

Superoxide dismutase is widespread in the human body, including skin and its appendages. Here, we focus on human skin copper/zinc superoxide dismutase, the enzyme that protects skin and its appendages against reactive oxygen species. Human skin copper/zinc superoxide dismutase resides in the cytoplasm of keratinocytes, where up to 90% of cellular reactive oxygen species is produced. Factors other than cell type, such as gender, age and diseased state influence its location in skin tissues. We review current knowledge of skin copper/zinc superoxide dismutase including recent studies in an attempt to contribute to solving the question of its remaining unexplained functions. The research described here may be applicable to pathologies associated with oxidative stress. However, recent studies on copper/zinc superoxide dismutase in yeast reveal that its predominant function may be in signaling pathways rather than in scavenging superoxide ions. If confirmed in the skin, novel approaches might be developed to unravel the enzyme's remaining mysteries.

## Introduction

Skin envelops the entire surface of human body and is daily exposed to environmental insults such as pathogens, injuries and ultraviolet (UV) radiation. Its important functions include regulation of body temperature, defense, and sensation as well as production of vitamin D. One of the skin's several defense mechanisms against environmental insults involves its structural organization: both skin layers, epidermis and dermis, contain cells, enzymes and other substances that play a critical role in defense. Several enzyme families contribute specifically to skin defense by scavenging pathogen-, injury- and UV radiation-derived molecules. The noxious molecules called free radicals can only be destroyed by the antioxidant activity of enzymes such as catalase, superoxide dismutase, peroxidase, and some supporting enzymes. We will focus here on human skin superoxide dismutase (SOD) highlighting, in addition to its best-known function of scavenging, further functions already shown in other models.

## ROS

**Figure 1:**
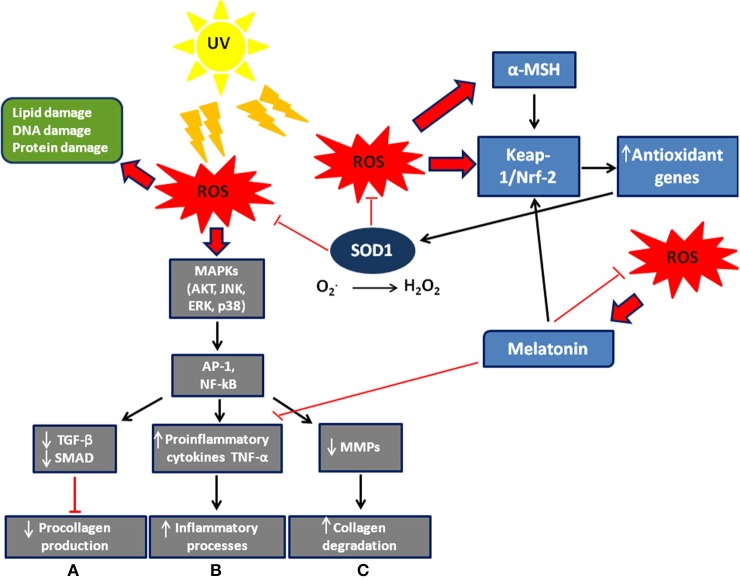
Scheme of ROS signaling/effects and defense system in the skin. Exogenous and endogenous agents of skin cells generate ROS, whose excessive levels can cause cell damage. ROS modulate MAP signaling pathways leading to activation of the transcription factor AP-1 (activator protein 1) and NF-kB (nuclear factor kappa-light-chain-enhancer of activated B cells). They also induce: **(A)** a decrease in procollagen synthesis via blocking TGF-β/SMAD signaling; **(B)** an increase in the inflammatory processes; **(C)** an increase in collagen degradation via synthesis of matrix degenerating MMPs. Furthermore, ROS allow nuclear factor Nrf2 to detach from its cytoplasmic inhibitor, keap-1, to translocate to the nucleus and activate transcription of antioxidant genes. Melatonin (8) and α-MSH (9) are also stimulated by ROS to activate Nrf2 dependent-pathways and then the expression of antioxidant genes. The Nrf2 pathway is activated not only in different skin cells such as keratinocytes and melanocytes, but also in fibroblasts (10). Modified by Sardy (11).

Free radicals have a single unpaired electron on the outer orbit. Derived from oxygen and also known as reactive oxygen species (ROS), they are generated by normal cellular aerobic respiration and are produced in mitochondria, endoplasmic reticulum, and peroxisomes, and are involved in several biochemical reactions that regulate fundamental cellular signaling pathways such as cell proliferation, apoptosis, and autophagy (1, 2). The energy created by their unstable configuration is freed through reactions with adjacent molecules, such as inorganic and organic substances, proteins, lipids and carbohydrates, and with key membrane molecules and nucleic acids (3–5). Oxygen radicals and other reactive species cause modifications in the amino acids of proteins, which frequently result in functional or structural changes of enzymatic proteins (6). They are able to induce covalent bonds with Kelch-like ECH-associated protein 1 (Keap1), a keratinocyte cytoplasmic protein that is normally linked to nuclear factor erythroid 2-related factor 2 (Nrf2), which, according to Dinkova-Kostova et al. (7), dissociates from Keap1 and transmigrates to the nucleus where it acts as a transcription factor and induces the production of antioxidant enzymes including copper/zinc SOD (Cu/Zn SOD) (Figure 1). ROS generation can also trigger autocatalytic reactions resulting in more free radicals that propagate the damage chain. These are inherently unstable and generally decay spontaneously. The superoxide anion, for example, is unstable and decays spontaneously in the presence of water to release oxygen and hydrogen peroxide (12).

There are many other exogenous factors, such as pollutants and UV that can induce ROS production. Excessive ROS production can lead to skin aging and development of skin cancer (13–15). Human skin is constantly exposed to three main types of UV [(16–18)^1^]. However, epidermis, by means of its self-protecting cells, keratinocytes, undergoes cornification. And among the proteins that make up the thickened cell envelope, small proline rich proteins, also known as stress-inducible proteins, are expressed that protect keratinocytes from ROS (19).

## Superoxide Dismutase

SOD is a highly conserved enzyme, that is abundantly expressed in the cytoplasm of aerobic organisms and plays a fundamental role in protecting cells from oxidative stress. It belongs to a family of enzymes that catalyze the dismutation of the superoxide radical (20, 21). These radicals are generated in many cellular processes (22) such as products of normal respiration and oxidative bursts from immune cells. There are various forms of SOD that incorporate different covalently bound substances (Mn, Zn, Cu, Fe) (23), and can inactivate both intra- and extra-cellular superoxides (20). Work on their subcellular localization in hepatocytes shows that Cu/Zn SOD (SOD1), which contains copper and zinc, may be found in the nuclei, cytoplasm, peroxisomes and lysosomes, but also in mitochondrial intermembrane space (24–26). Mn SOD (SOD2) contains manganese and it is predominantly found in the mitochondrial matrix (27). The third one, Cu/Zn extracellular SOD (SOD3), also contains copper and zinc and is secreted in the extracellular space (28). Superoxide radical anions are unstable molecules, commonly produced by aerobic metabolism. Oxidative stress, induced by the uncontrolled production of superoxide and its reaction products, is implicated in the development of pathologies including neurodegenerative diseases, premature aging, cancer, diabetes, and dermatitis. There is a clear increase in ROS generation of the skin after UV exposure (6). Epithelial cells and thymus-based fibroblasts can release antioxidant enzymes, especially under stress conditions (29). All mammalian cells express both mitochondrial Mn SOD and cytosolic Cu/Zn SOD, while the extra-cellular SOD high molecular weight isoform appears to be expressed only in specific cell populations (30, 31). Extra-cellular SOD was discovered by Marklund (30, 32) who showed that it is localized in the fluid and in the extra-cellular matrix of tissues; its role in cancer cells has been reviewed by Griess et al. (33) who suggested that extra-cellular SOD increase generated by ROS might work as a cancer suppressor (33). Immunohistochemistry has shown that EC SOD is also present in connective tissue. It is absent, however, in the thymus, stomach and skeletal muscle (34). Although it has been observed that extra-cellular SOD protects the extracellular space and the endothelial cell surface, its activity is low (32). These observations suggest that the enzyme can act both by a paracrine mechanism and on remote cells (35, 36). The hydrogen peroxide generated by SOD activity acts as an inhibitor of the enzyme. In this case the superoxide radical is not neutralized and inhibits the enzymes involved in subsequent reactions (e.g., catalase) (37–39).

Cu/Zn SOD is a stable 15.9 kDa homodimer. The dimerization is held by hydrophobic contacts (40) that reduce solvent accessibility and increase its stability (41). Each monomer contains two metals, a copper ion and a zinc ion, which together have either a structural or catalytic function. Initially Cu/Zn SOD was detected in the cytosol (24), in the outer membrane and/or in the intermembranous space between mitochondria and peroxisomes, which generate superoxide radicals (42). In fibroblasts, Cu/Zn SOD appears to be localized in peroxisomes (43). An important novel function of Cu/Zn SOD has been reported recently in yeasts and humans. In response to oxidative stress, high levels of H_2_O_2_ promote Cu/Zn SOD nuclear translocation and as a transcription factor the enzyme regulates the expression of oxidative resistance and repair genes (44). In addition, in yeasts, only a small amount of Cu/Zn SOD was shown to scavenge superoxides while the majority of Cu/Zn SOD mediated peroxide signaling (45). There are as yet few similar investigations on vertebrate cells including human; it would therefore be of great interest to confirm the data obtained in *Saccharomyces cerevisiae*. Actually, it has been reported that 25% of genes related to human degenerative pathologies overlap almost completely with those of yeast pathologies, thus allowing the study of homologous antioxidant response genes in much simpler eukaryotic organisms (46).

## Cu/Zn SOD and Skin

**Figure 2:**
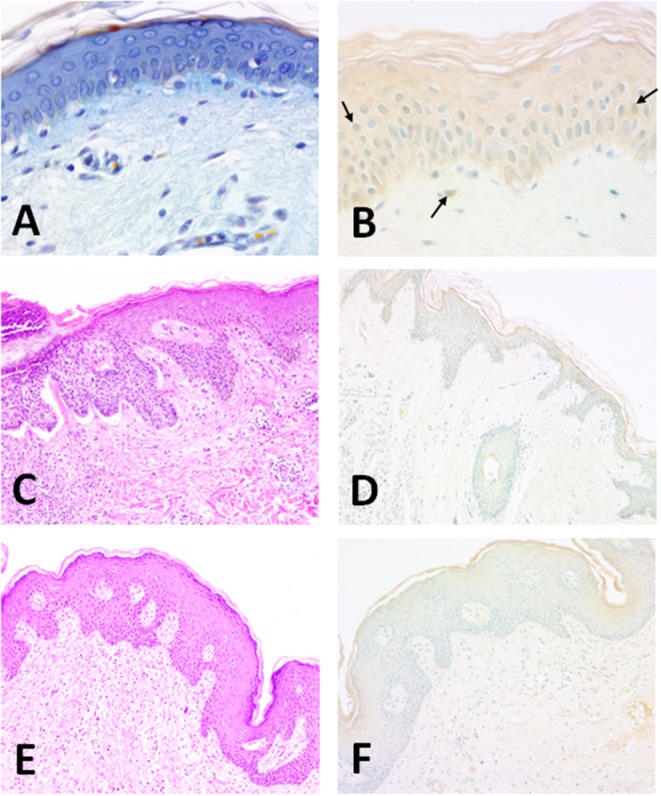
**Structure of the normal human epidermis** (man, aged 25; forearm skin). **(A)** Section of human skin showing the different layers of the epidermis. OPA trichrome staining (47). 40 x magnification; **(B)** The enzyme is clearly present in epithelial keratinocytes. Horseradish peroxidase development. Methyl green counterstaining allows a better identification of SOD1-immunoreactive nuclei (arrows). Specimens are subsequently evaluated by histo-densitometry of Cu/Zn SOD immunoreactive areas. 10x magnification. **Illustration of BCC** (man, aged 71; back, scapular skin). **(C)** Structure of the epidermis showing a nodular lesion in the dermis. Hematoxylin-Eosin. 10x magnification; **(D)** Densitometric measurements of Cu/Zn SOD for basal carcinomas were made at the level of the surface epidermis and in the tumor areas invaginated in the dermis. Faint staining of epidermis. Horseradish peroxidase development. Methyl green counterstaining. 10x magnification. **Illustration of SCC** (woman, aged 65; back, scapular skin). **(E)** Structure of the SCC-affected skin. Hematoxylin-Eosin. 10x magnification; **(F)** Densitometric measurements of Cu/Zn SOD in SCC-affected human skin were made at the level of the surface epidermis and in the tumor areas invaginated in the dermis. Epidermal staining is weaker than normal tissue. Horseradish peroxidase development. Methyl green counterstaining. 10x magnification.

In a recently published immunocytochemical study Altobelli et al. localized Cu/Zn-SOD in human skin in several conditions (Figures 2A–F). Some studies show that during the processes of natural aging and photo-aging the activity of SOD does not change in the skin, while that of catalase is increased in the epidermis and considerably reduced in the dermis (48). The activity of antioxidant enzymes seems to vary among cell types; for instance, fibroblasts have high levels of catalase, glutathione peroxidase and SOD compared to keratinocytes (43). In the skin, environmental direct contact-induced ROS production can cause aging, skin diseases, and cancer. Photo-aging depends on the degree of exposure and the type of skin. For example, individuals who live in warm environments are more exposed to light and are consequently prone to photo-aging (49). However, according to Hellemans (50), the seasonal activity of SOD in the *stratum corneum* does not seem to vary.

UV rays induce the synthesis of matrix metalloproteinases (MMP) in the skin, leading to the destruction of collagen (51) (Figure 1). The greatest effect is observed in photo-aging, where there is a greater activity of MMP and an increase in the accumulation of hydrogen peroxide, due to reduced dermal catalase. The accumulation of hydrogen peroxide changes the activity of mitogen-activated protein (MAP) kinases involved in the synthesis of pro-collagen and consequently induces aging (50, 52–54). *In vitro* studies of the effects of SOD on cellular antioxidant metabolism have shown its relationship with MMP and its consequent regulation of extracellular matrix degradation (55). These data were later confirmed in a study *in vivo* on a pig model. Clinical results of radiation-induced fibrosis have provided some evidence that Cu/Zn SOD could become an anti-fibrotic drug, its therapeutic effect relating to down-regulation of transforming growth factor beta 1 (TGF-beta1). In fact, SOD significantly reduces the expression of TGF-beta1 while an increase of TGF-beta1 expression is associated with fibrotic diseases (56).

Direct murine skin-exposure to various oxidative stresses requires a high antioxidant capacity to maintain a low oxidant balance (57). Measurements of enzymes and antioxidant substances present in human skin showed that the activity of SOD was higher in the epidermis than the dermis in both young and aging skin (48). These results were then confirmed by histo-densitometry and showed a reverse relationship between human cutaneous Cu/Zn SOD and aging, as well as a higher level in the male than the female (58). In addition, exposed skin expressed more of the enzyme than non-exposed skin (Figure 2B). Interestingly, it has been proved that carbazole induces ROS production in the human keratinocyte cell line, HaCaT. Carbazole is an aromatic heterocyclic compound so called because it contains one or more aromatic rings in its structure. It has been shown that when carbazole is present in human skin, for example in tattoo ink, prolonged exposure to sunlight resulting in photosensitized carbazole causes downregulation of antioxidant genes (hmox-1, keap-1, nrf-2, and bcl2) in HaCaT cells and of course an increase of ROS, with consequent apoptotic cell death (59). *In vitro* experiments with HaCaT (59, 60) provide important information on the physiology of SOD and oxidative stress-linked skin diseases. HaCaT cell lines have also been used to unravel an unexpected relationship of the NO/NOS system with Cu/Zn SOD. NO is thought to up-regulate Cu/Zn SOD expression that in turn might inhibit the mechanism of keratinocyte proliferation (61).

Thus, SOD protects human keratinocytes from UV-induced damage that includes aging caused by fragmentation of collagen and elastic fibers and activation of metalloproteases (62). A comprehensive review of mainly plant-derived natural antioxidants for human skin has been produced by Dunaway et al. (63): it has been reported that antioxidants such as green tea, vitamin C, vitamin E, CoQ10 and hydroxytyrosol could reduce the effect of UV radiation (64–69). In human skin, a single exposure to UV rays involves a transient reduction of SOD activity; however, after chronic UVB irradiation, activity of epidermal SOD is induced (6). Studies on mouse skin have shown that UVB lowers the level of Cu/Zn SOD, while Mn SOD is influenced by UVA. Seasonal alternation, however, does not cause a variation in SOD concentration in either exposed or non-exposed skin (50).

It seems that HaCaT cell lines become apoptotic in a dose-dependent way 24 h after radiation with 150 J/m^2^ of UVB and only 35 J/m^2^ of UVC (70). These negative effects are reduced in the body thanks to the activity of antioxidant enzymes.

## Basal Cell and Spinocellular Carcinomas

The skin is the organ generally most affected by tumors because it is the most exposed to environmental insults. The importance of SOD in the skin is not always clear, i.e., its expression may be influenced by cytokines (71).

The activity of SOD is variable: it is reduced in the presence of melanomas, epitheliomas, and carcinomas (72). Immunohistochemical studies have shown in fact that in carcinomas the activity of both Cu/Zn SOD and Mn SOD decreases (58, 73). EC SOD, however, is present both in the dermis and in the epidermis and its level of expression is seven times greater in the dermis (71). In another study both basal and spinous layers contained Cu/Zn SOD with a prominent expression in the upper epidermal layers (58) (Figures 2C,D). In addition, a weak intracellular distribution was observed throughout the human epidermis and especially in the *stratum corneum* (6).

Chronic exposure of the skin to sunlight promotes premature aging, reduces its immunological response to environmental antigens and is the main risk factor in the development of a variety of precancerous and malignant skin neoplasms due to the uncontrolled growth of keratinocytes and melanocytes (74–76). Although ultraviolet rays are responsible for tanning, the UVB component in particular can damage skin cell DNA (77). Damage to the DNA induces in turn the modulation of genes concerned with the activation of various signal transduction pathways, promoting tumor growth from initiated cells, which can grow into tumors because they divide much more rapidly than normal ones (78).

Skin tumors develop mainly in areas most exposed to the sun: face, ears, neck and scalp (79). Basal cell carcinoma (BCC) is particularly frequent and is the most common invasive skin carcinoma in humans, followed by spinocellular carcinoma (SCC). Melanoma is rarer, but better known and more dangerous. Malignant skin tumors develop relatively slowly and are largely treatable if detected early and then treated promptly (80). Immunohistochemical analysis of BCC and SCC revealed a reduction of immunoreactive Cu/Zn SOD and Mn SOD, indicating a UV-dependent depletion of antioxidant defenses; this reduction seems to be inversely proportional to the proliferation of malignant cells (81). In another study there was a noticeable increase in SOD activity at 24 h from exposure to UVB rays, with a significant increase in Cu/Zn SOD and a reduction in Mn SOD levels (82). Notably, it has been reported that melatonin has a protective action against UV radiation (UVR)-induced 8-hydroxydeoxyguanosine formation and depletion of antioxidative enzymes. The experiment used *ex vivo* human full-thickness skin exposed to UVR in a dose- (0, 100, 300 mJ/cm^2^) and time- (0, 24, 48 h post-UVR) dependent manner. Thus, melatonin plays a crucial role as a potent antioxidant and DNA protectant against UVR-induced oxidative damage in human skin (83).

Benign epithelial neoplasms are common and generally biologically harmless. These tumors, deriving from the multi-layered keratinized basal epithelium of the epidermis, hair follicles, and the ductal epithelium of the cutaneous glands, generally maintain the characteristics of their cells of origin. With regard to malignant tumors, the incidence of BCCs increases significantly in immunosuppressed patients and in those with congenital defects of DNA repair mechanisms and affects individuals over 45 years of age (84, 85). These tumors can take various forms and can affect every part of the body but most often the head. Approximately 33% patients a year develop another primary BCC. SCCs occur on light-exposed skin of elderly subjects. Except for lesions of the lower limbs, these tumors have a higher incidence among men. They are more aggressive than BCCs, expanding more rapidly and occasionally (<5%) metastasizing to nearby lymph nodes where they are generally profoundly invasive (85).

The main predisposing factor is exposure to UVR from the sun and consequent DNA damage; other factors include industrial carcinogens, chronic ulcers and draining osteomyelitis, old burn scars, ingestion of arsenical substances, ionizing radiation and (for the oral cavity) mastication of tobacco (86, 87). Sunlight, in addition to its effects on DNA, also seems to have a direct transient immunosuppressive effect on the skin, altering the normal immune-surveillance function of its Langerhans cells. SCCs, often preceded by preneoplastic lesions (actinic keratoses), are usually diagnosed and operated early when they heal in 95% of cases, otherwise they can invade the deep tissues and destroy the bone and cartilage, especially around the eyes, nose, and ear. Morphologically these epitheliomas are nodular, flat (cicatricial), sclerodermiform and pigmented, and may be ulcerated. Both BCCs and SCCs express Cu/Zn SOD enzyme mostly in the upper epidermis (Figures 2E,F). Furthermore, histo-densitomentric evaluation of these tumors has recently demonstrated increased human epidermal SOD activity with respect to dermal one, and more expression in human male skin than in female as well as more in young than old people (58).

## Conclusion

Cu/Zn SOD has long been known as an enzymatic protein, but morphological studies in skin began only in the late nineteen-eighties, first in pig and then in human (88, 89). The first observations allowed the localization of two types of SOD, the Cu/Zn-type and a Mn-type. However, these initial morphological studies were aimed at understanding the relationship between SOD activity and epidermal proliferation. Then, using cultured human keratinocytes, different roles were established for Cu/Zn SOD and Mn SOD. Cu/Zn SOD activity increases after irradiation as a reaction to ROS-generated oxidative stress, therefore acquiring an antioxidant function (89). Furthermore, it was suggested that cytokines IL-1 alpha and TNF-alpha produced by keratinocytes could mediate SOD recovery after the defense reaction. During the past decade understanding has changed, as described above: many other Cu/Zn SOD functions have been proposed; for example, the role of transcription factor and, with the help of the yeast, *S. cerevisiae*, and HaCaT cells, the function of oxidative signaling, which predominate over that of scavenging. Thus, Cu/Zn SOD crosses the nuclear membrane to join promoters and activate transcription of determinate signaling pathways. In fact, Cu/Zn SOD-containing cells show immune-positive nuclei as well as cytoplasm. It remains to be clarified why nuclear labeling by immunogold doesn't occur in Kobayashi's skin preparations (89). In another investigation, Cu/Zn SOD nuclear translocation was shown in yeast and human fibroblast (44). Additional evidence for the anti-oxidant role of this enzyme comes from work on skin atrophy and delayed wound healing in SOD-deficient mice which can be reversed with various anti-oxidants, such as syringaresinol, vitamin C derivatives and Palladium/Platinum nanoparticle mixtures (90–93). It would be useful if all these data could be confirmed in the human epidermis. Skin is a very complex biochemical laboratory. Many aspects of antioxidant enzymes have been discovered during the past 5 years but there are more to be elucidated before the definitive functions of Cu/Zn SOD and other antioxidant enzymes in human skin are fully understood.

## Author Contributions

VC and GA: conceptualization and design of the study. VC: drafting of original manuscript. VC, GA, and AB: literature reviewing. SV: editing and revising manuscript critically for intellectual content. GA, SV, AB, and VC: final approval of the manuscript as it has been submitted.

## Conflict of Interest

The authors declare that the research was conducted in the absence of any commercial or financial relationships that could be construed as a potential conflict of interest.

## Footnotes

^1^http://www.who.int/mediacentre/multimedia/en/
